# Circulating Hematopoietic (HSC) and Very-Small Embryonic like (VSEL) Stem Cells in Newly Diagnosed Childhood Diabetes type 1 – Novel Parameters of Beta Cell Destruction/Regeneration Balance and Possible Prognostic Factors of Future Disease Course

**DOI:** 10.1007/s12015-021-10250-7

**Published:** 2021-09-12

**Authors:** Milena Jamiołkowska-Sztabkowska, Kamil Grubczak, Aleksandra Starosz, Anna Krętowska-Grunwald, Magdalena Krętowska, Zuzanna Parfienowicz, Marcin Moniuszko, Artur Bossowski, Barbara Głowińska-Olszewska

**Affiliations:** 1 Department of Pediatrics, Endocrinology, Diabetology With Cardiology Division, Medical University of Bialystok, Białystok, Poland; 2 Department of Regenerative Medicine and Immune Regulation, Medical University of Bialystok, Białystok, Poland

**Keywords:** Type 1 diabetes, Beta cell function, C-peptide, HSC, Partial remission, Regeneration, Stem cells, VSEL

## Abstract

**Aims/Hypothesis:**

We aimed to evaluate hematopoietic stem cells (HSC) and very small embryonic-like stem cells (VSEL) mobilization to establish their role in residual beta cell function maintenance and partial remission occurrence in children newly diagnosed with type 1 diabetes.

**Methods:**

We recruited 59 type 1 diabetic patients (aged 6–18 years) monitored for 2 years, and 31 healthy children as a control group. HSC and VSEL levels were assessed at disease onset in PBMC isolated from whole peripheral blood with the use of flow cytometry. An assessment of beta cell function was based on C-peptide secretion. Studied groups were stratified on the basis of VSEL, HSC and/or C-peptide median levels in regard to beta cell function and partial remission.

**Results:**

Patients with higher stimulated C-peptide secretion at disease onset demonstrated lower levels of HSC (p < 0.05), while for VSEL and VSEL/HSC ratio higher values were observed (p < 0.05). Accordingly, after 2 years follow-up, patients with higher C-peptide secretion presented lower initial levels of HSC and higher VSEL/HSC ratio (p < 0.05). Patients with lower values of HSC levels demonstrated a tendency for better partial remission prevalence in the first 3 to 6 months after diagnosis.

**Conclusions:**

These clinical observations indicate a possible significant role of HSC and VSEL in maintaining residual beta cell function in type 1 diabetic patients.

**Graphical Abstract:**

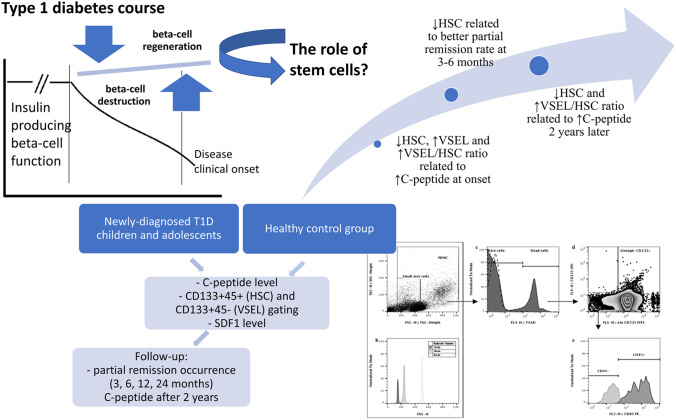

**Supplementary Information:**

The online version contains supplementary material available at 10.1007/s12015-021-10250-7.

## Introduction

Type 1 diabetes remains one of the most frequent autoimmune diseases in children and adolescents, with increasing morbidity observed in last decades [1]. The disease itself is characterized by selective, immune-mediated destruction of insulin-producing beta cells. Both historical and recent studies have demonstrated that at the time of diagnosis about 70–80% of beta cell mass is already damaged [2, 3]. Previously, it was believed that their progressive destruction leads to a total lack of insulin secretion in a short period of time [4]. Nowadays, however, we know that insulin-producing cell loss in type 1 diabetes does not seem to be linear and that remaining beta cell mass is maintained through spontaneous regeneration and stimulated recovery [5].

Velocity and intensity of the beta cell damage progression reported at the time of type 1 diabetes clinical diagnosis varies among patients. A rapid loss of beta cell function can be observed in some patients, in contrary to others experiencing partial remission (PR—a period of a good metabolic control with low daily insulin requirement and with reduced risk of severe hypoglycemia). Noteworthy, Joslin Medalist Study revealed that sustained residual beta cell function can still be detected even in patients with disease duration of 50 years [6]. Numerous potential factors have been described to be related to PR frequency, including: age, gender, presence of DKA, physical activity and other autoimmune diseases coexistence [7, 8]. To date, the underlying mechanisms of that phenomenon have not yet been fully explained and determining whether a particular patient will experience sustained beta cell function and/or PR is still challenging for clinicians.

Considering regenerative processes occurring parallel to islets destruction since the very beginning of the disease, increased interest in potential strategies promoting beta cell regeneration has been observed in recent years. Stem cells transplantation is expected to became a promising alternative to immunosuppressive therapy in a number of autoimmune disorders [9]. Results from in vitro studies and clinical trials involving the use of mesenchymal (MSC) and hematopoietic (HSC) stem cells provided significant data on possible effective application of these cells in type 1 diabetes treatment. In addition, chance for implementation of such therapeutic approach is supported by the easy availability of autologous material [10, 11].

Despite the increasing number of studies on stem cells role in various pathological states, to date, there are neither data on stem cell patterns occurring at type 1 diabetes clinical onset, nor evidence whether there is a link between these cell populations and pancreatic beta cells function in the course of the disease. Therefore, the aim of our study was to evaluate peripheral blood mobilization of HSCs and VSELs in pediatric patients newly diagnosed with type 1 diabetes in order to establish their role in insulin secretion maintenance and partial remission occurrence – most critical aspects affecting the future life of young type 1 diabetic patients.

## Research Design and Methods

### Patients

We recruited 59 patients newly diagnosed with type 1 diabetes in the Department of Pediatrics, Endocrinology, Diabetology and Cardiology Division (Medical University of Bialystok, Poland) who were then monitored in Outpatient Clinic for subsequent 2 years. All of the patients fulfilled type 1 diabetes diagnostic criteria according to ISPAD guidelines and received insulin intravenously at the beginning of therapy. Next, the treatment was changed to subcutaneous functional intensive insulin therapy (in the form of multiple daily injections or continuous subcutaneous insulin infusion) when the patient’s condition was stabilized. All patients were Caucasian, mean age was 11.3 years (in range from 6 to 18 years). Anthropometric data: gender, weight, height, BMI and BMI-standard deviation score (BMI-SDS) were analyzed. Exclusion criteria were: age below 5 years, diabetes of unknown origin, diabetes diagnosed before clinical symptoms occurred (as a screening among a family with a diabetes history), lack of satisfactory attendance to control visits, or transfer to a different medical center during the follow-up observation.

To determine the remaining endogenous insulin secretion, C-peptide measurements were performed at the disease onset and after 2 years of therapy. Patients were divided into groups depending on their median C-peptide levels at different time points. HbA1c and total daily insulin requirement (DIR) [U/kg/24 h] were assessed: at diabetes onset (the day of discharge from hospital), after 3 and 6 months, and at 1^st^ and 2^nd^ year in the follow-up, to establish patients’ metabolic control and to determine PR occurrence (HbA1c < 53 mmol/mol [< 7%] and DIR < 0.5 U/kg/24 h) [12]. For the purpose of our study we determined a “PR expected at onset”, as some of the patients achieved an insulin demand < 0.5 U/kg/24 h with normoglycaemia before the discharge from hospital. Additionally, 31 healthy, age- and gender-matched children were recruited as a control group. Full clinical characteristics of the study groups is presented in (Table 1).

**Table 1:** General characteristics of the study groups. Data are presented as mean ± standard deviation or median (interquartile range) for normally and non-normally distributed data, respectively. BMI, BMI-SDS – body mass index standard deviation score, DKA – diabetic ketoacidosis, DIR – daily insulin requirement, PR – partial remission

	Type 1 diabetic patients	Healthy control
Number of patients	59	31
Gender (boys/girls) [%]	42.4/57.6	40.6/59.4
Age (at onset) [years]	11.28 ± 3.15	12.4 ± 3.46
BMI [kg/m2]	16.78 ± 3.43	19.92 ± 4.45
SDS-BMI	-0.38 (-1.1 – 0.39)	0.46 (-0.41 – 1.15)
Type 1 diabetes onset:		
C-peptide, fasting [ng/dl]	0.54 (0.35–0.8)	1.72 (1.67–2.59)
C-peptide, 2 h stimulated [ng/dl]	1.21 (0.48–0.74)	
DKA [%]	28.8	
HbA1c [mmol/mol] / [%]	118.26 / 12.91	
DIR [IU/kg/24 h]	0.63 (0.48–0.74)	
PR expected at onset [%]	28.8	
Follow up		
PR 3 months [%]	49.2	
PR 6 months [%]	47.5	
PR 1 year [%]	27.1	
PR 2 years[%]	10.1	
C-peptide, 2 years [ng/dl]	0.44 (0.17–0.77)	
HbA1c, 2 years [mmol/mol] / [%]	53.4 / 7.04	
HbA1c, mean in follow-up [mmol/mol] / [%]	47.4 / 6.49	

### Laboratory Analysis

For laboratory analyses venous blood samples were collected. Samples from diabetic patients at the time of diagnosis were collected 3–5 days after subcutaneous insulin therapy initiation and successful recovery from diabetic ketoacidosis (DKA), if present. C-peptide measurements were performed at the disease onset (fasting and 2 h meal-stimulated) and after 2-year follow-up (fasting), by applying the electrochemiluminescence method (ECLIA) with the detection limit assessed as 0.01 ng/ml for the assay. Metabolic control parameter—HbA1c was determined by high-performance liquid chromatography at every time point of the therapy.

### Flow Cytometric Analysis of VSEL and HSC Populations

Peripheral blood was processed to obtain plasma and peripheral blood mononuclear cells (PBMC). PBMCs were obtained through gradient density centrifugation with the use of 1.077 g/ml Pancoll separating solution (PAN-Biotech). Isolated cells were suspended in freezing medium: 10% DMSO (Sigma-Aldrich) in fetal bovine serum (FBS; PAN-Biotech), and stored as viable cells in liquid nitrogen. Plasma samples obtained prior to gradient centrifugation were stored in -80 °C. In the separate studies we verified PBMC isolation influence on VSELs number and confirmed its suitability for the investigation of these cells when strict collection, isolation, storage and analysis protocols are followed.

PBMCs of type 1 diabetes and control group pediatric patients were subjected to immunostaining with fluorochrome-conjugated monoclonal antibodies, including: Lineage1 (CD3, clone SK7; CD14, clone MϕP9; CD16, clone 3G8; CD19, clone SJ25C1; CD20, clone L27; CD56, clone NCAM16.2) FITC, CD45 PE (clone HI30), CD133 APC (clone W6B3C1) (BD Bioscience). 2 × 10^6^ of PBMCs from each subject were incubated with selected antibodies and followed by washing steps with PBS. Subsequently, viability dye – 7 amino-actinomycin D (7AAD; BD Bioscience) was added into the samples to exclude dead cells from further analysis. Flow cytometric data acquisition was performed immediately after staining with the use of FACS Calibur flow cytometer (BD Bioscience). The obtained data were analyzed using FlowJo software (Tree Star Inc.). First, small cells of 2 to 6 μm were extracted from PBMC population on the basis of relative size (forward scatter, FSC), relative internal structure (side scatter, SSC) and size beads (2 to 6 μm beads; Life Technologies). 7AAD allowed to exclude 7AAD + dead cells from the analysis. Furthermore, gate was set on cells negative for all Lineage1 markers and positive for the marker of progenitor cells, namely CD133. Delineation of VSEL and HSC stem cell populations was based on differential expression of CD45 marker: CD45-CD133 + (VSEL) and CD45 + CD133 + (HSC). Proper gating strategy was established with the use of unstained and FMO (fluorescence minus one) controls (Suppl. 1).

### Immunoenzymatic Assessment of Cytokines in Plasma

Plasma from type 1 diabetes and control group subjects was used to evaluate concentration of SDF-1/CXCL12 (stromal cell-derived factor 1) concentration – factor participating in regulation of stem cells migration. SDF-1 levels were established in accordance with protocol included in the Human CXCL12/SDF-1 ELISA kit (R&D Systems). Data were acquired with the use of LEDETECT96 microplate reader (Labexim) set to 450 nm and wavelength correction included.

### Statistical Analysis

Statistical analyses were performed using Statistica 13.0 (Stat Soft Krakow, Poland). All continuous variables were tested for normal distribution by the Kolmogorov–Smirnov with Lillefors correction and Shapiro–Wilk tests. Descriptive statistics were calculated as either mean ± SD or median (IQR), respectively. Unpaired Student t test was used for normally distributed variables and Mann–Whitney U test was applied for samples non-normally distributed to compare the differences between two groups. When comparing more than two groups, the ANOVA Kruskal–Wallis test and the median test with post-hoc tests for multiple comparisons were used. To compare categorical variables between groups χ^2^ test with Yates correction was applied. Correlations between studied variables were assessed using Pearson correlation. The level of statistical significance was set at p < 0.05.

## Results

**Fig. 1:**
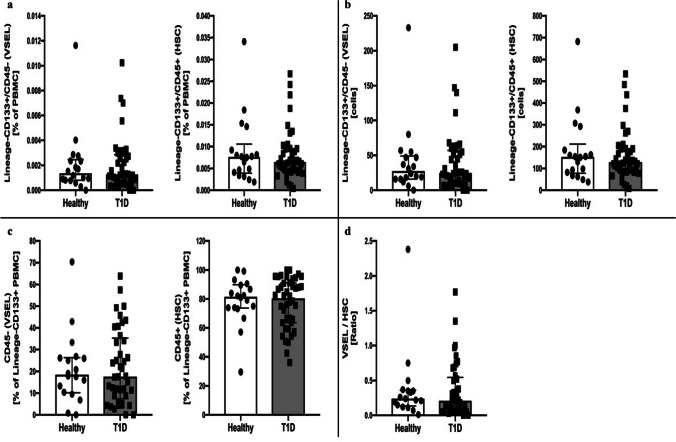
Analysis of VSEL and HSC levels in type 1 diabetes children and healthy control groups including frequency within PBMC (**a**), absolute number of these populations (**b**), distribution within Lineage-CD133 + progenitor cells (**c**), and VSEL/HSC ratio (**d**)

**Fig. 2:**
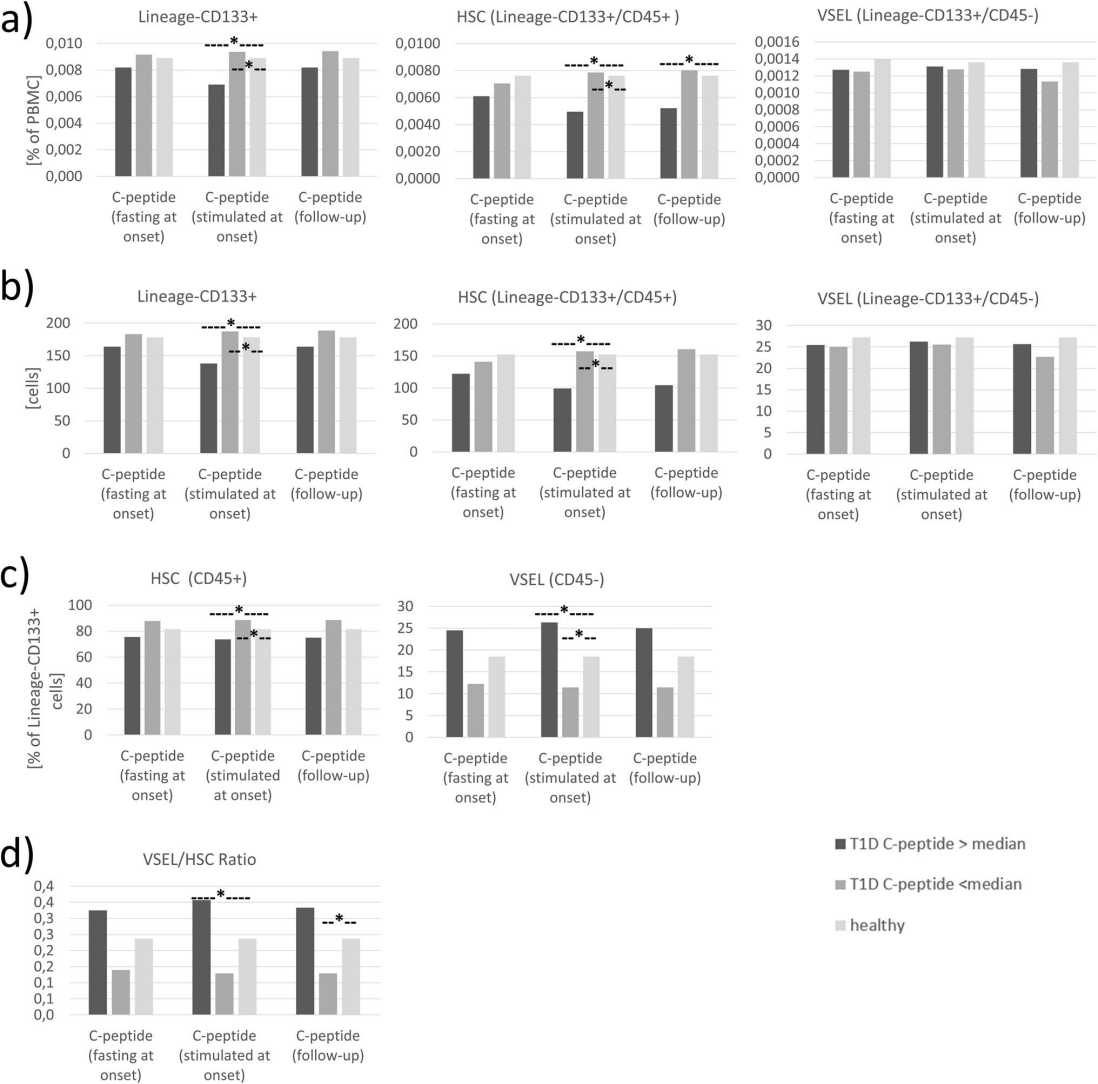
Investigation of differences in VSEL and HSC levels between pediatric patients with type 1 diabetes (T1D) divided on the basis of median C-peptide secretion and healthy controls. Analysis included VSEL and HSC frequency within PBMC (**a**), absolute number of these populations (**b**), distribution of VSEL and HSC within Lineage-CD133 + progenitor cells (**c**), and VSEL/HSC ratio (**d**). T1D groups were created on the basis of median C-peptide value (fasting/stimulated at onset and at follow-up) and corresponds to its level in specific study group. *—p < 0.05

Initial analysis of VSEL and HSC did not reveal statistically significant differences between newly-diagnosed diabetic patients and control group, regardless of whether frequency within PMBC or Lineage-negative CD133 + cells, absolute cell numbers, or VSEL/HSC ratio was analyzed. (Fig. 1) However, stratification of diabetic patients based on median C-peptide level allowed us to discover that patients with higher stimulated C-peptide secretion values at diabetes onset demonstrated lower levels of HSC (p < 0.05) when compared to lower C-peptide level group and to control subjects. In contrary, we did not observe significant differences in VSEL levels when analyzing frequency within PBMC and absolute numbers. However, distribution of VSEL within Lineage-negative CD133 + progenitor cells and VSEL/HSC ratio was found to be significantly higher in patients with higher stimulated C-peptide at the disease onset (p < 0.05). Lower levels of HSC (percentage and absolute number) and higher VSEL/HSC ratio were also found in patients with higher C-peptide secretion at 2^nd^ year of follow-up (p < 0.05) in comparison to lower C-peptide secretion group. (Fig. 2) (Suppl. 2) In correlation analysis significant negative correlation was found between stimulated C-peptide at diabetes onset and HSC level (r = -0.396, p < 0.05).

**Fig. 3:**
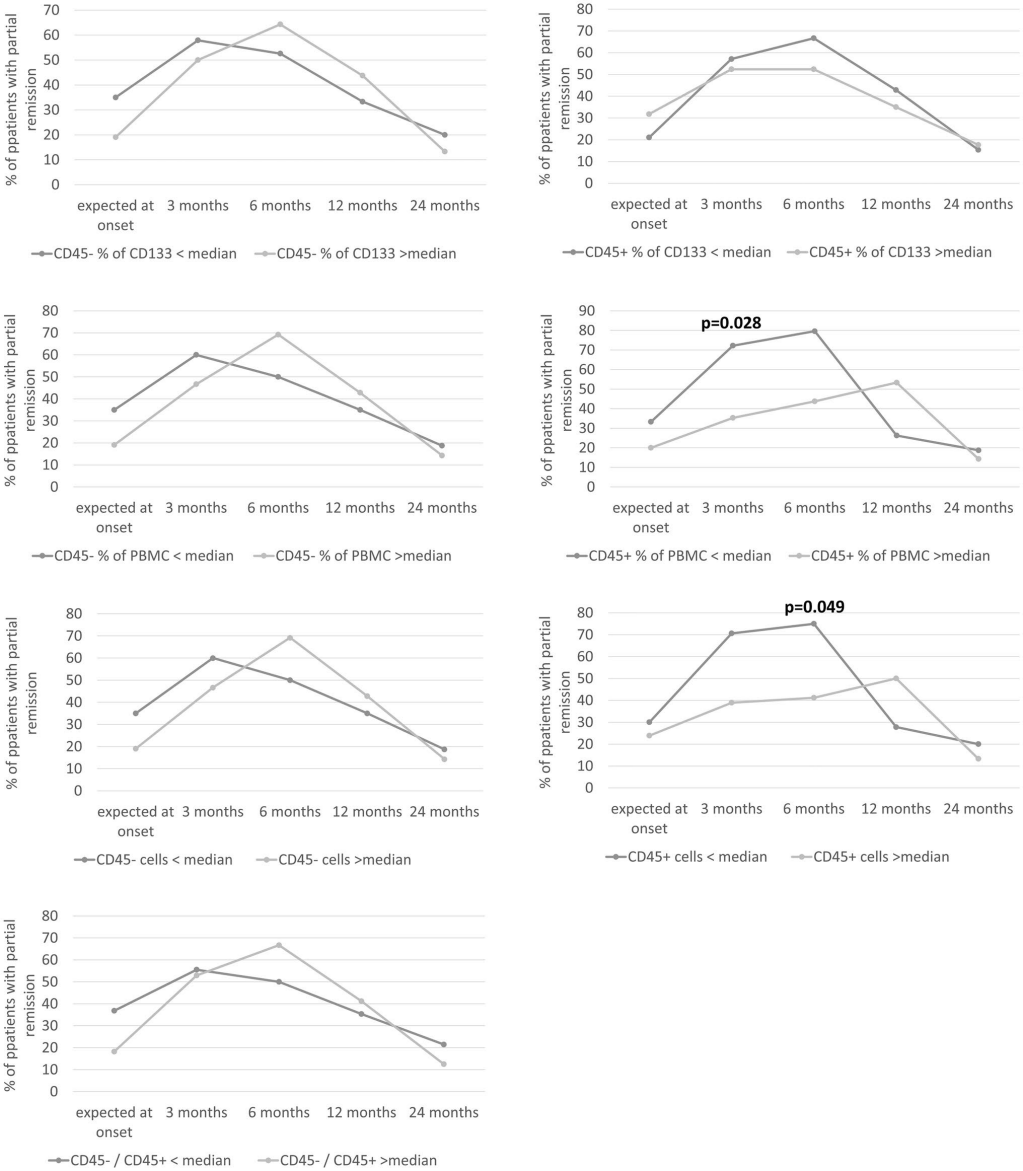
Assessment of connection between partial remission prevalence within patients with type 1 diabetes (T1D) [% of subjects at selected time point] and HSC/VSEL-related parameters (below or above median value of specific parameter). Analysis based on 2-year monitoring of T1D patients. χ^2^ p < 0.05 are presented

Next, in order to establish possible link between VSEL/HSC levels and PR among diabetic patients we divided them into two groups on the basis of VSEL or HSC median levels. Conducted analysis of PR occurrence among patients revealed that group with lower levels of HSC at the disease onset achieved higher PR frequency at 3^rd^ to 6^th^ month of the follow-up (p < 0.05). In reference to VSELs, its lower initial levels seemed to be associated with better remission prevalence only at 3^rd^ month, whereas patients with higher values for VSELs at the disease onset demonstrated higher frequency of PR at 6^th^ month. Noteworthy, at the final follow-up time point, despite slightly higher rates in groups with lower initial HSC and VSEL levels, frequency of patients with PR was comparable between two diabetic groups (no statistically nor clinically significant difference). (Fig. 3) Moreover, initial HSC levels correlated positively with HbA1c at 3^rd^ and 6^th^ month of the follow-up (r = 0.395 and r = 0.436, p < 0.05) and with DIR at 3^rd^ month (r = 0.528, p < 0.05).

**Fig. 4:**
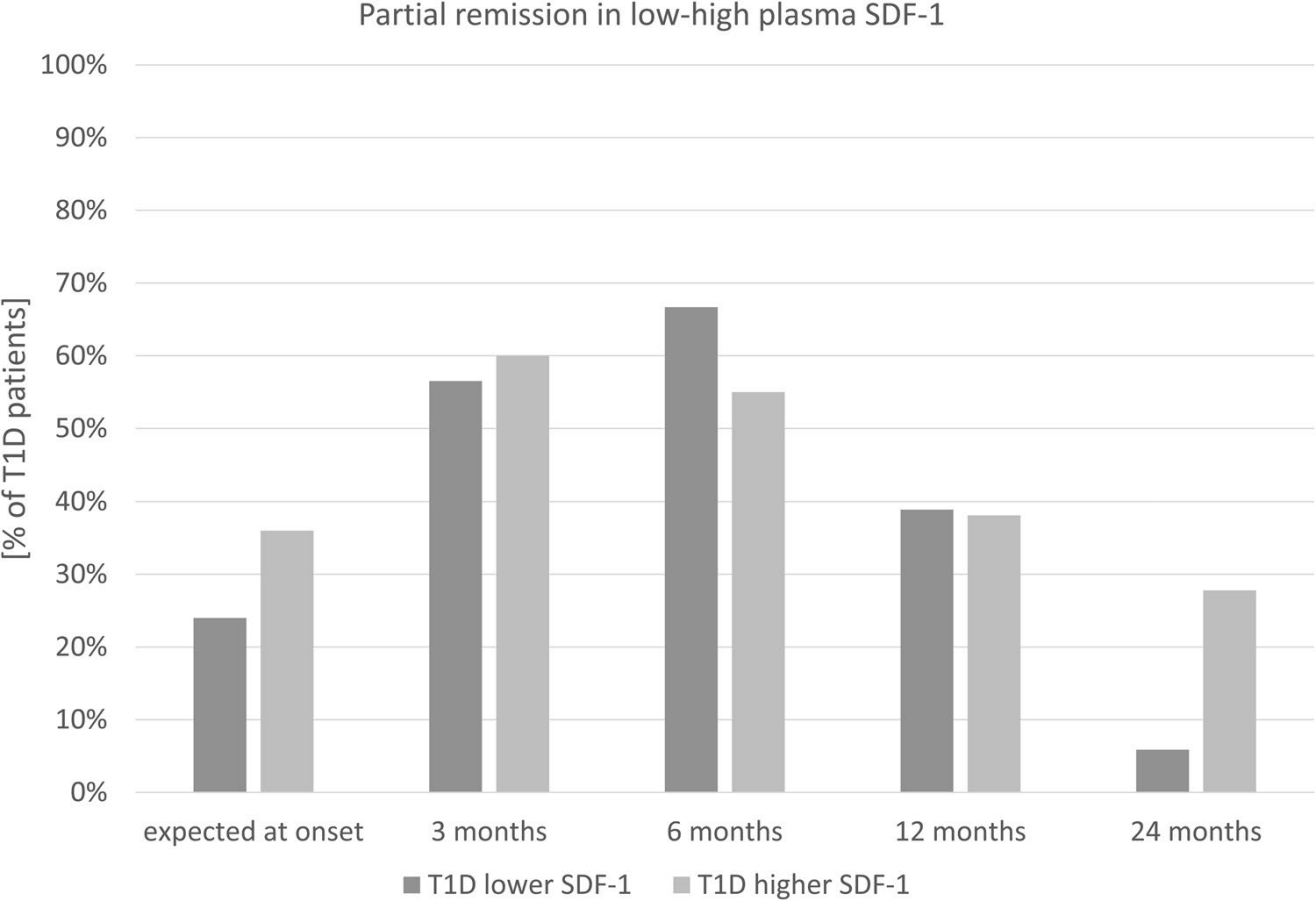
Comparison of partial remission prevalence within patients with type 1 diabetes (T1D) [% of subjects at selected time point] and SDF1-concentration (below or above median value)

In addition to VSEL and HSC levels analysis, we performed immunoenzymatic evaluation of SDF. We found no differences in plasma levels of SDF-1 between type 1 diabetic patients at the time of diagnosis and healthy control group. Stratification of type 1 diabetic patients on the basis of median C-peptide secretion revealed no significant differences in studied groups in reference to plasma SDF-1. However, PR occurrence changes in time in reference to median SDF-1 concentration demonstrated similar tendency as to median HSC/VSEL levels. (Fig. 4).

## Discussion

The current study was conducted in order to establish the selected stem cells populations role (VSEL and HSC) in newly diagnosed pediatric type 1 diabetic patients, taking into consideration both current beta cell function and its possible changes within years. The main finding of our study is that among diabetic patients HSC and VSEL levels and VSEL/HSC ratio analyzed at type 1 diabetes clinical onset demonstrated their possible application as predictors of C-peptide secretion and thus future beta cell function. Moreover, here we reported that the disease onset levels of HSCs and VSELs might be related to PR prevalence, especially in first 3 to 6 months following the diagnosis. Considering the data available, our results are the first to demonstrate such a link between HSC/VSEL levels and PR among pediatric type 1 diabetic patients. Reported HSC and VSEL levels were not associated with significant differences in SDF-1 plasma levels between healthy controls and T1D patients.

It is generally accepted that during gestation most of beta cells differentiate from adult stem cells or progenitor cells existing in pancreatic ducts, islets of Langerhans or in the bone marrow [13]. In postnatal period, beta cell formation was believed to physiologically originate from division and differentiation of those pre-existing differentiated cells, with the role of progenitor cells suggested to be mostly insignificant [14]. The expansion of beta cell mass dominates in early infancy with subsequent decline of beta cell replication rate [15] and significant beta cell turnover is reported to be limited to the first three decades of life [16]. Nevertheless, beta cell replication was observed to be stimulated by hyperglycaemia, fatty-acids, increased metabolic demand, incretin and placental hormones. Therefore, local pancreatic environmental factors (ex. during late pregnancy or after subtotal pancreatectomy) may lead to a beta cell hyperactivity with an increase of beta cell mass [17–19]. Beta cell replication was reported to be increased with islet inflammation, thus suggesting a compensatory proliferative response to autoimmune beta cell destruction and functional beta cell recovery of pre-existing beta cells [20]. Meier et al. presented strong evidence of inflammation-stimulated beta cell regeneration even in adults with either newly recognized type 1 diabetes or long-lasting disease [21, 22]. These data may explain the phenomenon of PR phase taking place just after type 1 diabetes diagnosis, that was demonstrated in our study in reference to stem cells activity.

HSCs isolated from bone marrow, cord blood or even peripheral blood after mobilization, are the most extensively characterized stem cells of mesodermal origin. Their most crucial function is the ability of self-renewal and differentiation into hematopoietic progenitor cells [23–25]. Regeneration capability was the main reason for the research considering HSC application in chronic autoimmune diseases treatment [26]. Studies on the possible contribution of HSCs in beta cell replacement through differentiation into insulin-producing cells revealed conflicting results. Ianus et al. presented some confirmatory data on HSC transdifferentiation into functioning pancreatic cells in mouse model [27], however other researches did not agree with these findings [28].

Although HSC potential to directly affect beta cell differentiation was not confirmed, HSCs may play an important role by protecting remaining beta cells in diabetic patients. Numerous studies presented evidence of pancreatic regeneration after bone marrow-derived cells transplantation with transplanted cells differentiating into endothelial cells of ductal and islet structures, accompanied by pancreatic cells proliferation and improved insulin production [29–31]. Moreover, transplanted HSCs were found to demonstrate a potential to suppress autoreactive immune cells, restoring islet cells tolerance and preventing T cell-mediated beta cell destruction [32]. A metaanalysis study showed a good clinical efficacy of stem cell-based therapy of diabetes with documented results of reduction of insulin dosage or even insulin independence [33]. In accordance, D’Addio et al. presented that autologous HSC transplantation could induce long-term insulin-free remission in patients recently diagnosed with type 1 diabetes [34].

Interestingly, in our study we did not find statistically significant difference in overall diabetic patients HSC levels in comparison to healthy individuals. Our finding remains somehow contrary to previous reports that describe impaired HSC mobilization in diabetic mice [35], as to reported potentially diabetes-related impairment of stem cell function [36]. However, our results support the view that potential “diabetic cell mobilopathy” [37] does not occur early in the disease course, but is rather expected to appear in a long-lasting diabetes [38]. Moreover, our data do not exclude functional impairment of studied stem cells but concentrate predominantly on changes in their distribution and correlation with diabetes-related parameters.

In fact, among diabetic patients lower HSC mobilization was strongly connected with better residual beta cell function, both at type 1 diabetes onset and in follow-up. At the moment, the pathways regulating observed phenomenon are difficult to explain as there are no similar studies, however our results clearly suggest an existence of relationship between circulating HSC and the severity of insulin-producing cells destruction. Therefore, we assume that evaluation of HSC level at type 1 diabetes onset might possess a predictive value for further beta cell function.

As far as HSCs demonstrate only multipotency, thus, limited variety of regenerated tissues, more attention is paid recently to pluripotent populations. One of the most recent examples of these could be very small embryonic-like stem cells (VSELs), described as possibly originating from pluripotent primordial germ cells that migrated into body organs during early embryonic development. VSELs can be isolated either from peripheral blood, cord blood or even adult organs [39], and importantly, apart from the self-renewal ability they can differentiate into cell lineages of three germ layers [40]. Noteworthy, during stress and tissue/organ injuries VSELs can be mobilized into circulation and migrate to affected areas [41–43]. Based on these data, VSELs are hypothesized to play a crucial role as a backup for specific progenitors within adult tissues, and thus, participate in regeneration processes of damaged organs [44]. VSELs present ability to differentiate into completely new pancreatic cells rather than to promote pre-existing islet restoration [45].VSELs relation to pancreatic cells damage was observed by Starzyńska et al., who reported their increased mobilization in patients with pancreatic cancer [46]. Huang et al. in other research presented evidence of VSELs mobilization into injured pancreas with their significant contribution to beta cell regeneration [47].

First attempts to use allogenic VSELs implantation in diabetes are dated back to 2015, with unequivocal evidence of their migration into diabetic pancreas and their further survival, however, the maintenance of cell function still remains to be answered [48]. Another study on animal model drew a hypothesis of VSELs role in hematopoiesis regulation, inter alia, through their transformation into CD45 + HSCs and further differentiation into major hematopoietic lineages [49]. Such demonstrated connections between VSELs and HSCs shed a new light on the possible implementation of stem cell-based therapies in diabetes management.

In our study, higher levels of circulating VSELs at type 1 diabetes clinical onset were observed in diabetic patients with better beta cell function based on the C-peptide secretion. The possible explanation of that finding might be the VSEL mobilization in response to pancreatic injury as a compensatory mechanism developing even prior the disease symptoms manifestation. Initial VSEL/HSC ratio was also observed to present statistically significant higher values in diabetic patients with higher C-peptide secretion both at diabetes onset and at 2^nd^ year of follow-up.

Interestingly, we have not found significant correlation between VSELs and studied beta cell function parameters, however observed phenomenon might be a part of a broader immune response pattern in patients with better C-peptide secretion or could be affected by not known preclinical disease duration in our patients.

In our opinion, VSELs together with HSCs could be considered a potential prognostic markers for residual insulin secretion, indicators of more advanced islet regeneration processes and suppressed impairment of beta cell function in those patients. Such findings also suggest an interesting cooperation of both systemic and local regenerative mechanisms involved in pancreatic beta cell regeneration. Moreover, VSEL mobilization might, therefore, also become a possible regenerative therapy in the early phase of type 1 diabetes.

Our data are the first to investigate the HSC and VSEL levels role in context of partial remission occurrence in the course of type 1 diabetes in pediatric patients. In children PR appears usually within first 3 to 6 months after the insulin therapy implementation and can be subsequently observed for months or even years, with mean duration of 6–9 months. However, its frequency declines with time [50]. Obtained results seem to be consistent with the current knowledge in the field, with lower HSC levels, higher VSEL levels and higher VSEL/HSC ratio associated with better remission rates in the follow-up, limited particularly to first 3 to 6 months after the diagnosis. Our observations suggest this time interval as a crucial moment for the PR phase development, and therefore emphasizing the possible role of HSC and VSEL levels as PR occurrence predictors.

Concluding, our study partially revealed a potential of stem cells in predicting future beta cell function in type 1 diabetes affected patients. Lower mobilization of HSC and higher mobilization of VSEL detected at the clinical onset of diabetes might become indicators of more efficient insulin production at that specific time point. Moreover, lower HSC level together with higher VSEL/HSC ratio seems to be a good predictor of less progressive loss of beta cell function. Patients with lower HSC and higher VSEL levels also demonstrated the tendency to better PR occurrence – especially in the crucial first months after diagnosis when PR develops.

We are aware, that this is the first study trying to find a link between HSC, VSEL and beta cell function in newly diagnosed T1D patients. There is, undoubtedly, a need to verify our results in further investigations. Furthermore, comprehensive research would be of a great importance to reveal possible cooperation of both local and systemic mechanisms associated with the observed phenomenon. However, we believe that the assessment of the studied parameters may play an important role in diabetology to achieve a reliable tool providing useful insight into the disease course and to selection of the patients who will benefit the most from the wide range of novel therapeutic options.

## Limitations of the Study

Some methodological limitations of the presented study should be noted. We did not include patients at age < 5 years. The reason of such a decision was a more severe disease course in those children, with observed extremely quick islet destruction described in literature. Considering that in our Department we observe only a few cases of newly recognized type 1 diabetes per year in this age group, and that in those patients possible underlying mechanisms might be different than in older children, we decided to keep our study group more homogenous. To assess beta cell function in follow-up we used fasting-C-peptide levels only, due to organizational and technical capabilities of our Outpatient Clinic. Finally, we are aware that a follow-up of 2-years is still a short observation time and for more complex conclusions it should be extended for next following years.

## Supplementary Information

Below is the link to the electronic supplementary material.

Supplementary file1 (DOCX 146 KB)

## Data Availability

The datasets analyzed during the current study are available from the corresponding author on reasonable request.
