# Supplementary material for: Circulating Hematopoietic (HSC) and Very-Small Embryonic like (VSEL) Stem Cells in Newly Diagnosed Childhood Diabetes type 1 – Novel Parameters of Beta Cell Destruction/Regeneration Balance and Possible Prognostic Factors of Future Disease Course
Source: Stem Cell Rev Rep. 2021 Sep 12;18(5):1657–67. doi: 10.1007/s12015-021-10250-7 (PMC9209363; doi:10.1007/s12015-021-10250-7)
Supplement: Supplementary file 1 — Supplementary file1 (DOCX 146 KB) [file 12015_2021_10250_MOESM1_ESM.docx]

**
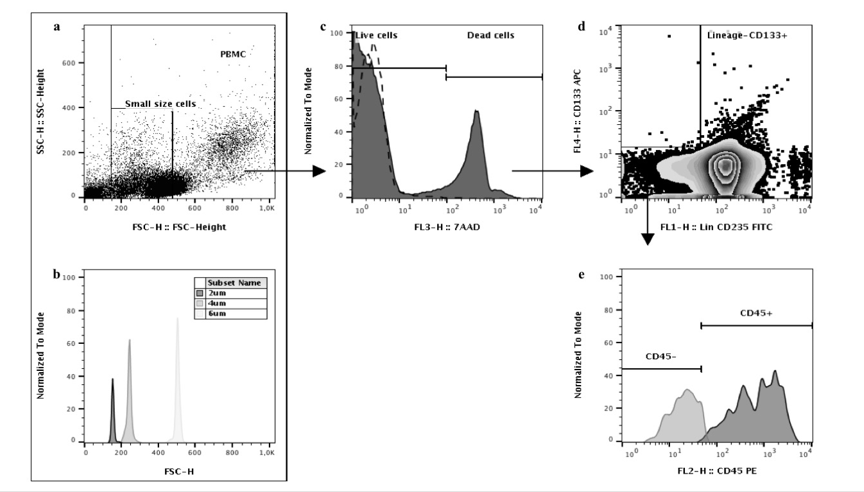
**

**Suppl.1.** Gating strategy of VSELs and HSCs. In the first step FSC/SSC properties (a) and sice beads (b) were used to distinguish small size cells of around 2 to 6μm. Prior to further analysis, dead cells were excluded (c), and subsequently CD133+ progenitor cells were gated out from Lineage (CD3+CD14+CD16+CD19+CD56+) negative population (d). Finally, Lineage-CD133+ progenitor cells were divided into CD45+ HSCs and CD45- VSELs (e).

| A) C-peptide fasting at onset | | | | | |
| --- | --- | --- | --- | --- | --- |
|  | Type 1 diabetes patients | | Healthy | Kruskal-Wallis  p-value | U-Mann-Whitney p-value |
|  | C-peptide > median | C- peptide <median |  |  |  |
| Lineage-CD133+  [% of PBMC] | 0.008  (0.007-0.009) | 0.009  (0.007- 0.012) | 0.009 (0.006-0.016) | n.s. | n.s. |
| Lineage-CD133+  [cells] | 163.87  (135.01-182.13) | 183.15  (137.74-242.33) | 178.09  (109.35-322.26) | n.s. | n.s. |
| Lineage-CD133+/CD45- (VSEL)  [% of PBMC] | 0.0013  (0.0009-0.0030) | 0.0013  (0.0007-0.0025) | 0.0014  (0.0008- 0.0024) | n.s. | n.s. |
| Lineage-CD133+/CD45+ (HSC)  [% of PBMC] | 0.006 (0.005-0.008) | 0.0070 (0.005-0.010) | 0.008  (0.004-0.009) | n.s. | n.s. |
| Lineage-CD133+/CD45- (VSEL)  [cells] | 25.45 (17.09-59.45) | 25.02  (13.50-50.75) | 27.23  (16.23-47.17) | n.s. | n.s. |
| Lineage-CD133+/CD45+ (HSC)  [cells] | 122.09  (91.57-170.19) | 140.97 (94.07-205.46) | 152.39  (82.03- 184.15) | n.s. | n.s. |
| CD45- (VSEL)  [% of Lineage-CD133+ cells] | 24.50  (12.0-36.4) | 12.25  (6.06-33.3) | 18.50  (10.4-26.1) | n.s. | n.s. |
| CD45+ (HSC)  [% of Lineage-CD133+ cells] | 75.5  (63.6-88.0) | 87.75  (66.7-93.9) | 81.50  (73.9-89.6) | n.s. | n.s. |
| VSEL/HSC Ratio | 0.325  (0.136-0.571) | 0.139  (0.065-0.500) | 0.236  (0.154-0.353) | n.s. | n.s. |
| B) C-peptide stimulated at onset | | | | | |
| Lineage-CD133+  [% of PBMC] | 0.007  (0.003-0.008) | 0.009  (0.007-0.016) | 0.009  (0.006-0.016) | **0.0082** | **0.002311** |
| Lineage-CD133+  [cells] | 138.11  (64.36-165.70) | 187.33  (143.29-310.57) | 178.09  (109.35-322.26) | **0.0082** | **0.002311** |
| Lineage-CD133+/CD45- (VSEL)  [% of PBMC] | 0.0013  (0.0009-0.0030) | 0.0013  (0.0006-0.0032) | 0.0014  (0.0008-0.0024) | n.s. | n.s. |
| Lineage-CD133+/CD45+ (HSC)  [% of PBMC] | 0,005  (0.002-0.006) | 0.008  (0.006-0,0105) | 0.008  (0.004-0.009) | **0.0044** | **0.000883** |
| Lineage-CD133+/CD45- (VSEL) [cells] | 26.22 (17.26-59.45) | 25.55  (11.16-63.32) | 27.23  (16.23-47.17) | n.s. | n.s. |
| Lineage-CD133+/CD45+ (HSC) [cells] | 99.01  (38.14-122.09) | 157.20  (121.61-210.37) | 152.39  (82.03-184.15) | **0.0044** | **0.000883** |
| CD45- (VSEL)  [% of Lineage-CD133+ cells] | 26.30  (17.9-40.7) | 11.45  (4.35-27.8) | 18.50  (10.4-26.1) | **0.0280** | **0.013333** |
| CD45+ (HSC)  [% of Lineage-CD133+ cells] | 73.7  (59.3-82.1) | 88.55  (72.2-95.7) | 81.50  (73.9-89.6) | **0.0280** | **0.013333** |
| VSEL/HSC Ratio | 0.357  (0.217-0.688) | 0.129  (0.046-0.385) | 0.237  (0.154-0.353) | **0.0256** | n.s. |
| C) C-peptide in follow up | | | | | |
| Lineage-CD133+  [% of PBMC] | 0.008  (0.006-0.009) | 0.009  (0.007-0.020) | 0.009  (0.006-0.016) | n.s. | n.s. |
| Lineage-CD133+  [cells] | 163.87  (119.72-177-85) | 188.53  (135.59-396.60) | 178.09  (109.35-322.26) | n.s. | n.s. |
| Lineage-CD133+/CD45- (VSEL) [% of PBMC] | 0.0013  (0.0004-0.0030) | 0.0011  (0.0006-0.0022) | 0.0014  (0.0008-0.0024) | n.s. | n.s. |
| Lineage-CD133+/CD45+ (HSC) [% of PBMC] | 0.005  (0.004-0.007) | 0.008  (0.006-0.014) | 0.008  (0.004-0.009) | n.s. | **0.045487** |
| Lineage-CD133+/CD45- (VSEL) [cells] | 25.65  (8.66-59.59) | 22.68  (11.16-44.67) | 27.23  (16.23-47.17) | n.s. | n.s. |
| Lineage-CD133+/CD45+ (HSC) [cells] | 104.28  (83.23-137.74) | 160.60 (111.63-270.00) | 152.39  (82.03-184.15) | n.s. | **0.045487** |
| CD45- (VSEL)  [% of Lineage-CD133+ cells] | 25.00  (12.0-40.5) | 11.45  (5.33-33.3) | 18.50  (10.4-26.1) | n.s. | n.s. |
| CD45+ (HSC)  [% of Lineage-CD133+ cells] | 75.0  (59.5-88.0) | 88.55  (66.7-94.7) | 81.50  (73.9-89.6) | n.s. | n.s. |
| VSEL/HSC Ratio | 0.333  (0.136-0.682) | 0.129  (0.056-0.500) | 0.237 (0.154-0.353) | n.s. | **0.012767** |

**Suppl.2** Investigation of differences in VSEL and HSC between healthy controls and pediatric patients with type 1 diabetes (T1D) to reveal possible differences between studied groups in context of VSEL and HSC frequency within PBMC and absolute number of these populations. T1D groups were created on the basis of C-peptide secretion median value and corresponds to its level in certain groups. A) fasting C-peptide at onset, B) stimulated C-peptide at onset and C) C-peptide in follow-up. All results are presented as median and quartile range. P values <0.05 are shown.
